# Opposite role of Bax and BCL-2 in the anti-tumoral responses of the immune system

**DOI:** 10.1186/1471-2407-4-54

**Published:** 2004-08-24

**Authors:** Gwenola Bougras, Pierre-François Cartron, Fabien Gautier, Stéphane Martin, Marité LeCabellec, Khaled Meflah, Marc Gregoire, François M Vallette

**Affiliations:** 1 UMR 601 INSERM/UN, (Equipe 2), 9 quai Moncousu 44035 Nantes Cedex 01. France; 2 UMR 601 INSERM/UN, (Equipe 4), 9 quai Moncousu 44035 Nantes Cedex 01. France; 3 Clinique Universitaire de Neurochirurgie, Hôpital G&R Laennec, CHU Nantes. Boulevard Jacques Monod 44093 Nantes Cedex 01. France

## Abstract

**Background:**

The relative role of anti apoptotic (i.e. Bcl-2) or pro-apoptotic (e.g. Bax) proteins in tumor progression is still not completely understood.

**Methods:**

The rat glioma cell line A15A5 was stably transfected with human *Bcl-2 *and *Bax *transgenes and the viability of theses cell lines was analyzed in vitro and in vivo.

**Results:**

*In vitro*, the transfected cell lines (huBax A15A5 and huBcl-2 A15A5) exhibited different sensitivities toward apoptotic stimuli. huBax A15A5 cells were more sensitive and huBcl-2 A15A5 cells more resistant to apoptosis than mock-transfected A15A5 cells (pCMV A15A5). However, *in vivo, *in syngenic rat BDIX, these cell lines behaved differently, as no tumor growth was observed with huBax A15A5 cells while huBcl-2 A15A5 cells formed large tumors. The immune system appeared to be involved in the rejection of huBax A15A5 cells since i) huBax A15A5 cells were tumorogenic in *nude *mice, ii) an accumulation of CD8+ T-lymphocytes was observed at the site of injection of huBax A15A5 cells and iii) BDIX rats, which had received huBax A15A5 cells developed an immune protection against pCMV A15A5 and huBcl-2 A15A5 cells.

**Conclusions:**

We show that the expression of Bax and Bcl-2 controls the sensitivity of the cancer cells toward the immune system. This sensitization is most likely to be due to an increase in immune induced cell death and/or the amplification of an anti tumour immune response

## Background

Glioblastoma Multiforme (GBM) are the most common and aggressive tumors of the central nervous system (CNS) [1]. Current treatments (e.g. chemo-and radiotherapy) have been relatively unsuccessful and no significant improvement in the prognosis has been recorded over the last 20 years [1]. Cancer cells are capable of inducing a specific immune response against the tumor and this property has been used in an attempt to design new therapeutic strategies [2,3]. In particular, anti-tumor vaccination strategies using known tumor-associated antigens or whole tumor extracts have been developed over the last few years [2,3]. However, a growing body of evidence has recently shown that tumors are capable of suppressing anti-cancer immune responses by the induction of tolerance, anergy or by selective killing of immune cells, thereby preventing their destruction by the immune system [4,5]. Dead cells have been also widely used as a source of tumor antigen but contradictory results have been reported on their effect on tumor growth [6]. We [7] and others [8,9] have shown that apoptotic bodies are capable of inducing a long-lasting and efficient immune response against tumors whereas others have suggested that necrotic cells could be anergic and tolerogenic [10,11]. Thus, the ability of dead cells to generate an immune response against a tumor could be associated with the nature of the death inducer used and/or the modus operandi of cell death (i.e. necrosis vs. apoptosis). Cytotoxic T lymphocytes (CTLs) and natural killer cells (NK), the major actors of the immune surveillance, have the ability to induce cell death by apoptosis mainly through two mechanisms: the death receptor pathway (i.e. CD95/Fas/APO-1, TRAIL) or the cytotoxic granules (i.e. perforin/granzyme pathway) [4,12]. Activation of death receptors appears to be sufficient to induce the cytosolic activation of caspases, the main proteolytic enzymes of apoptosis, in some tumors (class I) while, in class II tumors, amplification of the death signal occurs through mitochondrial activation of caspases [13].

Proteins of the BCL-2 family play a major role in the control of apoptosis both *in vitro *and *in vivo *in the latter pathway [14]. These proteins can be divided into anti-apoptotic proteins such as Bcl-2 and pro-apoptotic proteins such as Bax [14]. Inhibition of apoptosis through the overexpression of Bcl-2 promotes oncogenesis as demonstrated in some follicular B-lymphomas [15] while, on the other hand, the loss of Bax function has been associated with tumor progression and a bad prognosis in colon and gastric tumors having a microsatellite mutator phenotype [16]. We have recently observed that the expression of a gain of function variant of Bax can be associated with a longer survival in GBM patients [17]. Note that the expression of the anti-apoptotic molecule Bcl-2 and that of the pro-apoptotic Bax increased in parallel in low grade to high grade tumors of glial origin, suggesting that Bax and Bcl-2 could play an antagonistic but essential role in these tumors [18]. One of the most powerful mechanisms of control of tumor growth is exercised by the immune system. The immune surveillance hypothesis suggests that potentially dangerous cells could be eliminated through induction of cell death [3].

Although the CNS is usually considered an immune privileged site [5], specific cellular immune responses against tumoral antigens have been achieved in some animal models [19,20]. In GBM, the absence of Bax protein is compensated by an increased expression of Bak, another multidomain pro-apoptotic protein, which also maintains the immune-induced cell death [21]. Thus, manipulation of the expression of the BCL-2 family members could also be involved in the sensitivity of glial tumors to the immune system.

We have tested this hypothesis by establishing cell lines, which stably express transgenes encoding either for human *Bax *or *Bcl-2 *in a rat glioma model and analyzed the effects of these transgenes on the *in vitro *and *in vivo *growth of these cell lines.

## Methods

### Reagents

Unless specified, all reagents used in this study were from Sigma (St Quentin-Fallavier, France). Monoclonal anti-human Bax antibody (clone 4F11) was from Immunotech (Villepinte, France) and monoclonal anti-human Bcl-2 antibody (M 0887) was from Dako (Trappes, France); antibodies against rat Bcl-2 or Bax were respectively from Oncogene (Ab5) (Fontenay sous Bois, France) and from Pharmingen (13456E) (Le Pont de Claix, France). The fluorogenic peptide Ac-DEVD-AMC was from Bachem (Voisins les Bretonneux, France) and the lactate dehydrogenase (LDH) activity was measured using the Cytotox 96^® ^assay from Promega (Charbonnières, France) as described previously [17,18,21].

Experimental research on animals have been conducted according to recommendations of the French National Ethics committee, and are in compliance with the HelsinkiDeclaration.

### In vitro transfection, proliferation and induction of apoptosis

The rat glioma cell line, A15A5, was obtained from the European Collection of Animal Cell Culture (Valbonne, France). The cell line was maintained in RPMI-1640 (Invitrogen, Cergy-Pontoise, France) supplemented with 10% heat-inactivated FCS (Eurobio, Les Ulis, France), 100 μg/ml streptomycin, 100 U/ml penicillin and 2 mM L-glutamate in a 5% CO_2 _air-humidified atmosphere at 37°C. Plasmids encoding for human Bcl-2 or Bax were subcloned into pRcCMV (Invitrogen) as described by the manufacturer. A15A5 cells were transfected with either 2 μg pCMV vector, pCMV Bcl-2 or pCMV Bax. Plasmid DNA was introduced into 10^6 ^cells by electroporation (GenePulser, BioRad, Yvry sur Seine, France) using 200 V/cm and 250 μF. Transfected cells were selected and cloned in a medium containing neomycin (250 μg/ml) for several weeks before clonal dilution.

Apoptosis was induced by a short UV-treatment. Both untreated and UV-treated cells were cultured for a further 24 h under serum-free conditions. Cell death was also induced with FasL (0.5 μg/ml; a gift of Dr P. Saas EPI 119, Besançon, France), doxorubicin (doxo; 2 μM), staurosporine (STS; 1 μM), Na-Butyrate (NaB; 10 mM) or serum deprivation (d-serum) for 3 days. A MTT assay was used according to the manufacturer's instructions (Promega) to determine *in vitro *cell proliferation of transfected A15A5 cells.

### Tumor and cell extracts and Western blots

A15A5 transfected cells (10^5 ^cells) or tumors established in rats or mice were homogenized vol./vol. in RIPA buffer (PBS containing 1% NP-40, 0.5% Na-deoxycholate, 0.1% SDS, 10 nM PMSF, 10 nM aprotinin, 1 nM Na-orthovanadate). After several passages in a 2 ml glass Dounce homogenizer, the homogenates were centrifuged at 4°C at 13,000 g for 30 min. The resulting supernatants were assayed for protein concentration using the Bradford technique prior to analysis on 15% SDS-PAGE. Western blots were performed as described earlier [21], using primary antibodies anti-Bcl-2 (1 μg/ml), anti-Bax (2 μg/ml) and actin (0.5 μg/ml). The antibodies bound to Immobilon-P (Millipore, France) were detected by enhanced chemiluminescence (Amersham, Aylesbury, UK) using a second peroxydase-labelled antibody. The amount of immunoreactive protein was quantified using IP-Lab Gel Program (Signal Analytics, Vienna, USA) after scanning with an Imager (Q-Biogene, Strasbourg, France).

### Animal experiments

Inbred BDIX rats and Swiss *nude *mice were purchased from Iffa-Credo (L'Abresle, France) and were housed under standard conditions in our laboratory. huBax and huBcl-2 as well as pCMV A15A5 cells were injected into the brain of BDIX rats weighing between 200 and 240 g. All animal procedures were performed with approved protocols and in accordance with published recommendations for the proper use and care of laboratory animals. Briefly the rats were anaesthetised with an intraperitoneal injection of pentobarbital (50 mg/kg) and positioned in a stereotactic head frame. Aseptic surgical techniques were used to open the scalp in the midline and to expose the frontal and temporalis bones. A 1.0 mm aperture for implanting tumor cells was drilled through the skull. The stereotaxic position of this injection site was 2.5 mm anterior to the bregma and 2.0 mm to the right of midline. 10^4 ^tumor cells were implanted stereotactically at a depth of 3.0 mm into the cerebral parenchyma using a 10 μl Hamilton syringe with a 26-gauge needle. The volume injected was 5 μl and the hole was sealed with sterile bone wax. Alternatively, rats were injected *sc *with 10^5 ^cells into the hindlimbs and tumor growth was monitored every week by measuring the volume of the growing tumors. Swiss *nude *mice were treated similarly except that 10^4 ^cells were injected subcutaneously.

In order to evaluate the implication of huBax A15A5 cells preventive anti-tumoral treatment, we designed a protocol consisting of three *sc *injections of 3.3 × 10^4 ^huBax A15A5 cells 15, 10 and 5 days before injection of pCMV or huBcl-2 A15A5 cells. As a control PBS, A15A5 cell oncolysate, or apoptotic bodies derived from Na-Butyrate (NaB)-treated A15A5 cells as previously described [7]. Briefly, apoptosis was induced *in vitro *by a 10 mM NaB treatment in subconfluent A15A5 cultures. When signs of apoptosis were observed under a microscope (changes in cell morphology, detachment from dishes, chromatin condensation as viewed with Hoechst 33342) apoptotic bodies were collected, centrifuged and conserved at -80°C prior to use. Typically, 250 μg apoptotic bodies were mixed with 5 mg/ml BCG and injected subcutaneously three times over 15 days. As a control we used oncolysates obtained after several cycles of rapid freezing/ thawing of A15A5 cells and the lysates was injected together with 5 mg/ml BCG to the animals as above. On day 0, 5 days post-treatment, four groups of rats were *sc *challenged with 10^5 ^pCMV or huBcl-2 A15A5 cells. Tumor growth was evaluated daily over 60 days.

### Immunohistochemical analysis of tumor cell injection site

Immunochemical analysis was performed as on 12 μm brain frozen sections. Briefly, sections were fixed with 4% paraformaldehyde in PBS for 30 min at room temperature. Endogenous peroxydase activity was inhibited by a treatment with 0,3% H_2_O_2 _in methanol for 20 min. The sections were incubated overnight at 4°C with anti-rat CD8 (hybridoma supernatant, Ox 8) diluted 1 in 2 in 1% BSA in PBS, then a secondary antibody coupled to peroxydase was added. The staining was revealed with an AEC substrate.

### Flow cytometry determination of intratumoral immune population

Tumors were resected and minced into 1–2 mm^3 ^pieces, which were incubated in extraction buffer (30 U/ml hyaluronidase; 500 U/ml DNase, 0.01% w/v collagenase in PBS) at room temperature for 45 min and under constant agitation. The cell suspension was filtered through a sterile grid and washed three times with RPMI and maintained overnight in RPMI before analysis. For the phenotypic analysis, monoclonal antibodies obtained from Pharmingen raised against the following molecules were used: CD3 (556970; cl. 1F4), CD4 (554835, cl ox35), CD8 (554854, cl. Ox 8), CD161 (555006, cl. 10/78), CMH I (22301 D), OX 41/ CD172 (552297) and OX 62 (555010). Monoclonal antibodies raised against granzyme B (GrB, cl. 2C5/F5) was obtained from Chemicon (France) and Fas (AF 126) from R&D Systems (Lille, France). Cells were incubated with the primary antibodies for 30 min at 4°C and washed twice in PBS + 0.1% BSA. For the intracellular detection of GrB, cells were first fixed in 4% paraformaldehyde for 10 min at room temperature, washed with PBS + 0.1% BSA, then permeabilized with 0.1% saponin then incubated with the anti-GrB antibody for 30 min. The secondary antibody was then added for 30 min at 4°C and the cells washed 3 times with PBS + 0.1% BSA. Cells were analyzed on FACScalibur (Becton Dickinson, Le Pont de Claix France) using Cell Quest Pro software. A total of 5000 cells were counted in each experiment.

## Results

### Charaterization of rat glioma cells transfected with human Bax or Bcl-2 transgenes

**Figure 1:**
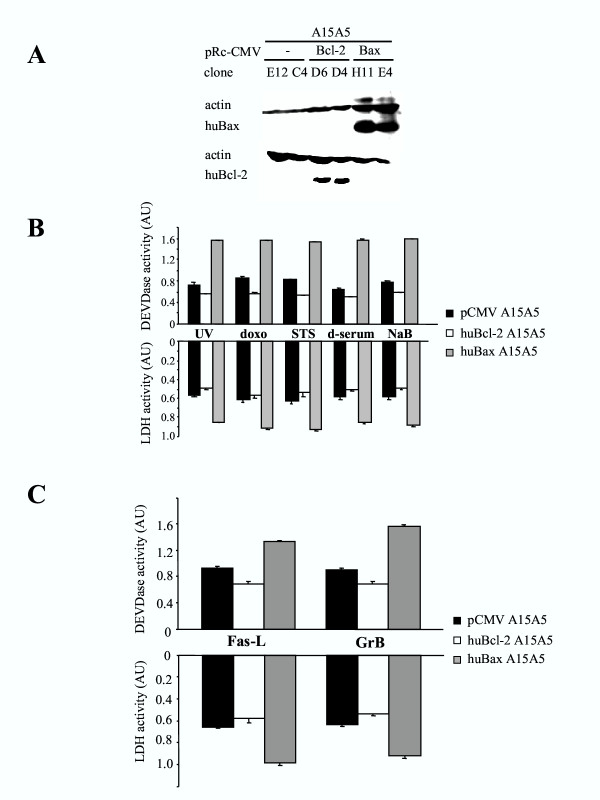
**A. **Western blot analyses of human Bcl-2 and Bax transgenes expression in stable A15A5 transfectants. Two clones per transfection with pCMV (E12 and C4), pCMV-Bcl-2 (Bcl-2: D6 and D4), pCMV-Bax (Bax: H11 and E4), which expressed approximately the same amount of trangenes were selected to be used in this study. **B. **The effect of huBcl-2 and huBax on the sensitivity of A15A5 cells to apoptosis, transfected cells was analysed after incubated with various cell death inducers as described in materials and methods. Apoptosis was determined by measuring the specific DEVDase and LDH activities after treatment of the cells with doxorubicin (doxo), staurosporine (STS), Na-Butyrate (NaB), serum deprivation (d-serum) or UV irradiation (UV). S.D. was calculated from 3 different experiments using 2 independent clones for each transgene. **C. **Cell death was induced by FasL and GrB. A15A5 cells were treated with FasL (0.5 μg/ml) or were transfected with a plasmid encoding for human GrB (200 μg plasmid) as described in **21**. In the latter case, we verified by FACS that similar amounts of human GrB were expressed in all transfected clones (data not shown). S.D. was calculated from 3 different experiments using 2 independent clones for each transgene.

A15A5 cells transfected with the pCMV vector, human *Bax *or *Bcl-2 *transgenes, were obtained as described in materials and methods. Two different clones were used in each experiment. The expression of the transgenes was monitored by immunoblot analysis using antibodies specific for human Bcl-2 or Bax and, as shown in figure 1A, transfections of the rat glioma cell line with human transgenes were efficiently achieved. Note that the overexpression of Bax did not induce apoptosis in the A15A5 cells, suggesting that the different clones selected expressed sublethal amounts of Bax.

To examine the effect of the different transgene expression to cell death, we studied their sensitivity toward different inducers of apoptosis using both drugs such as doxo (20 ng/ml) and STS (20 μM) or treatments such as d-serum, NaB (10 mM) or a short (1 min) UV irradiation. Cell death was monitored and quantified by measuring the activity of caspase 3 (namely the cleavage of the peptide Ac-DEVD-AMC) and the activity of the cytosolic enzyme LDH released into the culture medium (see materials and methods). As shown in figure 1B, when compared to pCMV A15A5 cells, huBax A15A5 cells were more sensitive to cell death in all cases. Conversely and as expected, huBcl-2 A15A5 cells were more resistant to all death inducers. Note that the resistance of pCMV A15A5 cells to apoptosis was already high, suggesting that these cells were naturally resistant to apoptosis. Nevertheless, these results suggest that human Bax and Bcl-2 transgenes were functional in the rat glioma A15A5 cells.

The immune system exerts its anti-tumoral surveillance mainly through cell death induced by CTLs and NK [12]. These cells use different effectors to mediate apoptosis in target cells: the death receptor mechanism such as the FasL/Fas receptor system or the perforin/GrB cytotoxic pathway [12]. The ligation of the death ligands to their receptors initiates cell death by the activation of the intracellular initiator caspase 8, which in turn can induce apoptosis either through the direct activation of caspase 3 in type I cells or by using mitochondria as an obligatory amplifier of the death signal in type II cells [4]. Cytolytic granules function through the serine protease GrB, which activates apoptosis mainly through the mitochondrial pathway [12]. We investigated the *in vitro *response to externally added FasL or by transient transfection of pCMV GrB, huBax A15A5 and huBcl-2 A15A5 cells as described previously for human glioma cells [21]. As shown in figure 1C, the expression of Bax sensitized the A15A5 cells to apoptosis induced by both FasL and GrB while apoptosis was inhibited by the presence of Bcl-2 under the same conditions. Incidentally, the fact that huBax or huBcl-2 transfection modulated apoptotic sensitivity toward FasL suggests that the A15A5 cells belong to the type II group.

**Figure 2:**
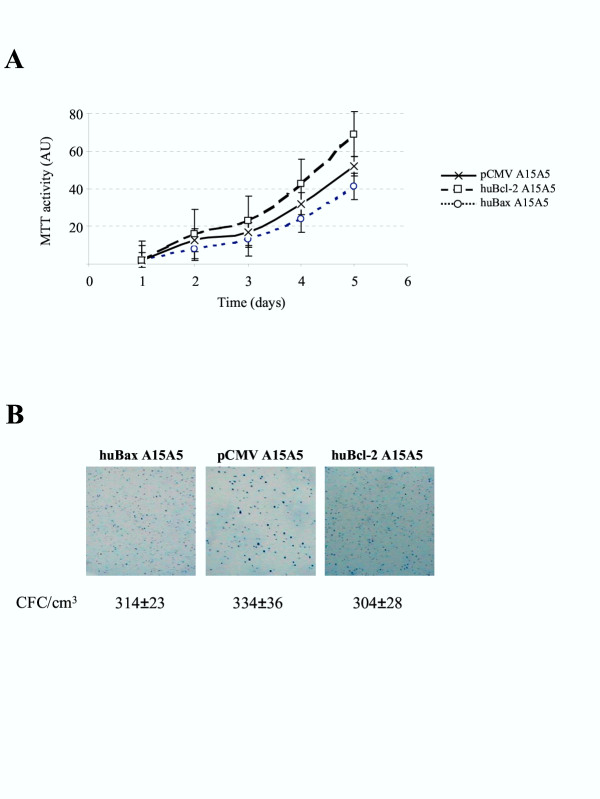
**A. ***In vitro *proliferation of transfected A15A5 cells was determined using a MTT assay. S.D. were calculated from 3 different experiments using for each, one of the 2 different clones for pCMV, Bcl-2 or Bax transfected cell lines. **B. **For the clonogenicity assay: 400 cells were plated into 6-well plates and colonies formed were counted as described in **21**. Data were given as number of CFU (colony forming units) with S.D. calculated from 3 independent clones for each transgene.

Bcl-2 expression has been associated with retardation in cell cycle entry [22] and as such we examined the proliferation rate of the different transfected cells using a MTT assay as described in material and methods. All transfected cells appeared to have a similar doubling time, which means that the expression of human Bax or Bcl-2 transgenes did not affect their proliferative capacity *in vitro *(figure 2A). Similarly, no significant differences in the effect of the transfection of pCMV, huBcl-2 or huBax A15A5 in A15A5 cells on the clonogenicity of the glioma cells were observed as shown in figure 2B.

### Tumorigenicity of Bax and Bcl-2 transfected cells

**Figure 3:**
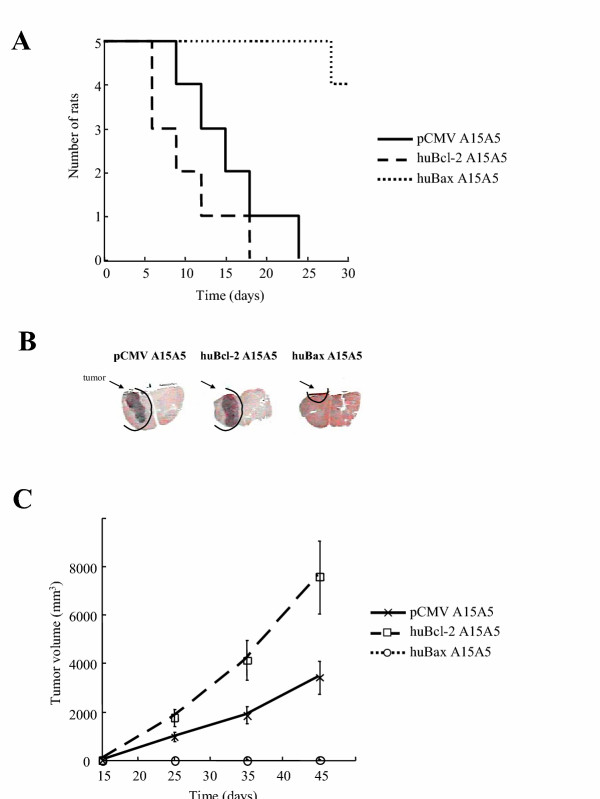
**A. **Growth of pCMV, Bcl-2 and Bax clones in BDIX syngenic rats. 10^5 ^cells from each clone were injected *ic *and rat survival was quantified using a Kaplan-Meier analysis. The curve illustrated is representative of 3 independent experiments. Each point represents the mean growth of 6 rats (3 rats per clone). **B. **The macroscopic observation of the tumors induced by the *ic *injection of huBcl-2, pCMV and huBax A15A5 cells into rat brains. Rat brains were stained with hematoxylin. **C. **Subcutaneous growth was followed by measuring the tumoral volume every 10 days after the injection of the cells. The curve illustrated is representative of 3 independent experiments. Each point represents the mean growth of 6 rats (3 rats per clone).

To determine the influence of Bax and Bcl-2 on tumoral growth *in vivo*, 10^4 ^pCMV, huBcl-2 or huBax A15A5 cells were delivered intracranially (ic) into syngenic BDIX rats as described in materials and methods. The survival rates of the different groups of rats were different as the median survival for the group of rats which had received huBcl-2 A15A5 and pCMV A15A5 cells were respectively 10.2 and 15.6 days, a difference, which was highly significant (*P *= 0.0086) (figure 3A). On the other hand, 80% of rats, which were injected with huBax A15A5 cells, had a disease-free survival of at least 30 days (figure 3A). It should be noted that 20% of the death, which occurred after injection of huBax, huBcl-2 or pCMV-A15A5 cells appeared to be due to operation-induced traumatisms since sham-operated animals gave similar results (**data not shown**). Brain sections from rats injected with huBcl-2 or A15A5 cells were histologically examined at the time of their death or after 30 days for rats injected with huBax A15A5 cells. As illustrated in figure 3B, brain sections from rats injected with huBax A15A5 cells showed little or no tumoral growth contrary to that observed in rats injected with pCMV or huBcl-2 A15A5 cells. This result suggested that tumoral growth was severely impaired in huBax A15A5 tumors but stimulated in huBcl-2 A15A5 tumors when compared to pCMV A15A5 tumors.

To gain information about the kinetics of tumoral growth, cells were subcutaneously (s.c.) injected into the hindlimbs of syngenic BDIX rats and the volume of the tumors evaluated every 10 days. As shown in figure 3C, results similar to that obtained with i.c. experiments were observed as no or little growth was observed with huBax A15A5 tumors whereas huBcl-2 A15A5 tumors exhibited a faster growth than pCMV A15A5 tumors.

**Figure 4:**
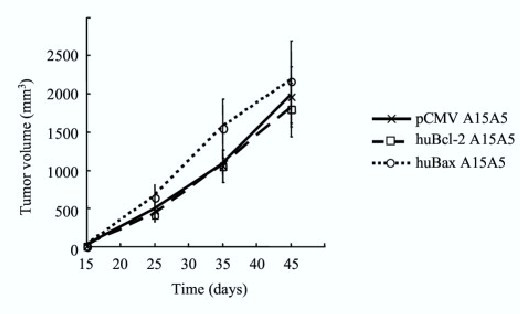
Growth of pCMV, huBcl-2 and huBax A15A5 cells in Swiss *nude *mice. 10^4 ^cells from each clone were injected *sc *and tumor growth was estimated every week by measuring tumor volume. The curve illustrated is representative of 2 independent experiments. Each point represents the mean of the growth of 6 mice (3 mice per clone). (□) pCMV; (○) Bax; (●) Bcl-2.

To test the involvement of the innate immunity in the control of proliferation, the different types of transfected cells (10^5^) were inoculated s.c. into Swiss *nude *mice (see materials and methods). Tumor growth in the mice was measured for a period of 45 days (figure 4), tumors developed rapidly with similar kinetics for all the A15A5 transfected cells including huBax A15A5 cells. Immunoblot analysis of Bax and Bcl-2 in tumors did not reveal any changes in the expression of Bcl-2 between the established tumors and the huBcl-2 A15A5 cells (**data not shown**). This result showed that the growth of the huBax A15A5 was not due to the induction of rat Bcl-2 expression during tumoral growth in the Swiss *nude *mice.

### Accumulation of CD8+ T cells in huBax A15A5 tumors

**Figure 5:**
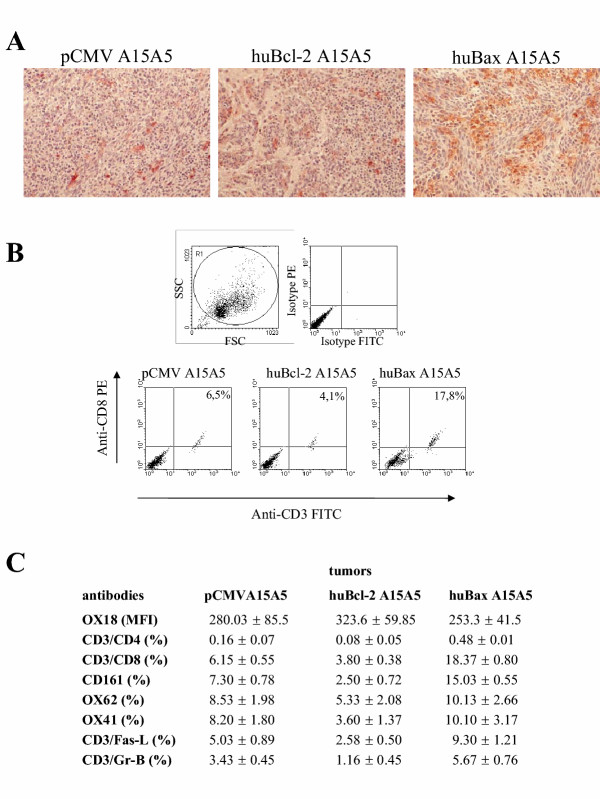
**A. **Qualitative analysis of CD8+ infiltrating lymphocytes by immunohistochemistry. **B. **Flow cytometric analyses of intra-tumoral CD8+ CTLs. Dot plots show the expression of CD3+ and CD8+ cells in the dissociated tumors. The percentages indicate the double positive cells for the three types of tumors. Results are representative of three independent experiments. **C. **Phenotypes of the intra-tumoral cells by flow cytometry. Tumors were treated as described in materials and methods. Results presented here are the mean of 3 independent experiments.

The latter result suggested that immune-induced apoptosis could be involved in the control of tumoral progression of A15A5 cells in BDIX rats, a feature partially lost in athymic mice. To assess local anti-tumoral response, we first investigated the presence of the CD8 marker in the different rat brain sections (figure 3B). The immunochemical analysis revealed a significant number of infiltrating CD8+ cells in huBax A15A5 tumors whereas huBcl-2 A15A5 or pCMV tumors showed very few CD8+ cells (figure 5A). Moreover, we noticed that necrotic tissue was absent, thus excluding a non-specific recruitment of lymphocytes due to an inflammatory process.

Next, we also quantified the accumulation of CD8+ cells by flow cytometry and examined the phenotypes of the infiltrating immune cells in *ic *tumors for the presence of intra-tumoral lymphocytes, monocytes and dendritic cells or the loss of the major histocompatibility (MHC) class I molecules. Tumoral cells were dissociated and enzymatically treated as described in materials and methods. We observed a significant increase in double positive cells CD3/CD8 in huBax A15A5 tumors (~17%) compared to huBcl-2 (~4%) and pCMV (~6%) tumors (figure 5B). An in-depth analysis of these CD3/CD8 cells (figure 5C) showed that only the lymphocyte T markers and the NK marker CD161 were significantly increased in huBax A15A5 tumors. On the other hand, no differences were found in the expression of class I MHC among the different tumors. The latter result suggested that T-cell mediated immunity could be involved in the rejection of the huBax A15A5 cells in syngenic rats. However, NK cells could play an auxiliary role in this process as suggested by the results obtained in *nude *mice.

### huBax A15A5 cells and A15A5 apoptotic bodies confer a protection against pCMV A15A5 cells and to a lesser extent against huBcl-2 A15A5 cells

The A15A5 rat glioma cells are highly immunogenic in the syngenic host [23], this immune response could eradicate tumors prone to apoptosis such as huBax A15A5 cells without affecting the viability of the cells resistant to apoptosis such as the pCMV or the huBcl-2 A15A5 cells. We have previously shown that apoptotic bodies derived from cells obtained from a rat colon carcinoma were a source of anti-tumoral antigens [7,24]. It is conceivable that the enhanced rate of cell death observed in huBax A15A5 tumors *in vivo *could generate a stronger immune response to pCMV or huBcl-2 A15A5 tumors.

**Figure 6:**
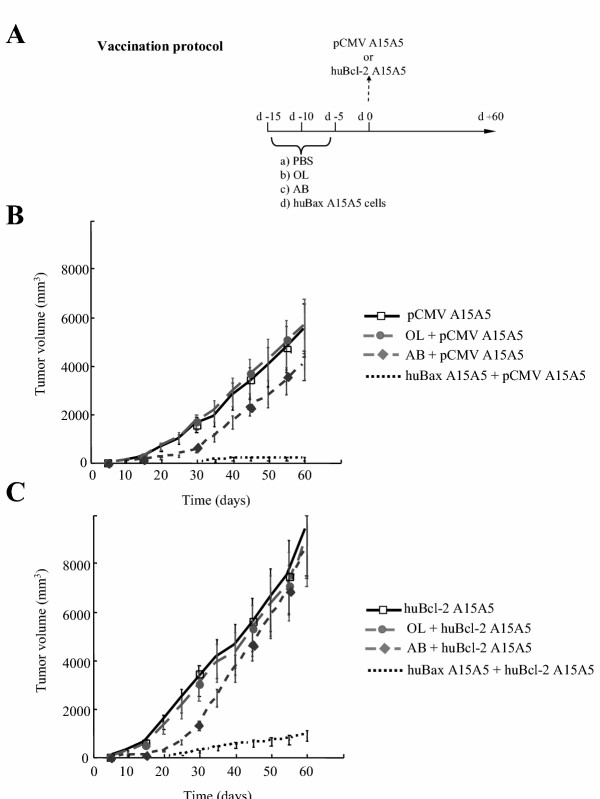
**A. **Vaccination protocol for the anti-tumoral immune response induced by huBax A15A5 cells, apoptotic bodies or oncolysates to pCMV or huBcl-2 A15A5 cells in rats. Rats were pretreated with PBS, oncolysate (OL) or apoptotic bodies (AB) from pCMV A15A5 cells or huBax A15A5 cells, 15, 10 and 5 days before being challenged with pCMV or huBcl-2 A15A5 cells. **B. **Comparison of tumor growth of pCMV A15A5 cells injected into syngenic BDIX rats. Four groups of 6 BDIX rats were treated as in **A **then on day 0 were challenged with 10^5 ^pCMV A15A5 cells injected *sc *and tumoral growth was followed for an 60 days. Data presented are illustrative of 3 independent experiments. **C. **Comparison of tumor growth of huBcl-2 A15A5 cells injected into syngenic BDIX rats. Four groups of 6 BDIX rats were treated as in **A **then on day 0 were challenged with 10^5 ^huBcl-2 A15A5 cells injected *sc *and tumoral growth was followed for an 60 days. Data presented are illustrative of 3 independent experiments.

To address this question, we compared the efficacy of an anti-tumoral immune response induced by huBax A15A5 cells to that observed with apoptotic bodies or oncolysates obtained from A15A5 cells (see protocol in figure 6A). Apoptotic bodies were generated from A15A5 cells treated with 10 mM NaB as described earlier [7] and their molecular characterization will be published elsewhere (Bougras et al. in preparation). BDIX rats were vaccinated with these apoptotic bodies using as a control A15A5 cell oncolysates or PBS (cf. materials and methods). The rats were then challenged either with pCMV A15A5 cells (figure 6B) or huBcl-2 A15A5 cells (figure 6C). As shown in figure 6B, the tumoral growth of pCMV A15A5 cells was reduced after the apoptotic body treatment although the effect was limited in amplitude and in time. On the other hand, no effect on tumor growth was observed after a similar treatment with A15A5 oncolysates (compared figure 6B with figure 3A). Interestingly, the treatment with apoptotic bodies triggered a weaker response to huBcl-2 A15A5 cells, which grew rapidly in animals treated with apoptotic bodies or oncolysate (figure 6C). As shown in figure 6B, rats that had received huBax A15A5 cells, then challenged with pCMV A15A5 cells developed small palpable tumors, which did not progress after 35 days post-challenging (i.e. pCMV A15A5 cell proliferation was abolished after 35 days by a huBax A15A5 cell pretreatment). Note that huBcl-2 A15A5 cell proliferation was reduced but not abolished in these rats (figure 6C). These results suggested that rats that had received huBax A15A5 cells were specifically protected against tumor growth and that the overexpression of Bcl-2 could not completely overcome this protection.

## Discussion

Tumor recurrence after surgical resection is often observed in GBM patients and additional chemo-or radiotherapy has not been shown to substantially improve survival in these patients [1]. In animal models, cancer vaccines have been shown to procure an appropriate immune response, to be highly specific and to favor tumor rejection [25]. In the case of CNS tumors, intensive research has been performed in the field of immunotherapy since the discovery that the CNS could not be regarded any longer as a completely immunologically privileged site [25]. Phase I studies have demonstrated the feasibility and the safety of this approach in human gliomas (see for example [26]). However, although some promising results have been obtained in preclinical studies, so far most clinical attempts have been disappointing. This setback in the application of immunotherapy is, however, not restricted to CNS tumors and new strategies are now being elaborated to enhance the efficiency of this approach. Others and we have observed that apoptotic bodies, the entities derived from apoptotic cells, could be a source of « new » anti-tumoral antigens and as such could be a source of potent tumor vaccines [7-9]. However, the nature of the cell death program, which gives an appropriate anti-tumoral immune response remains controversial [6].

Apoptosis is thought to be critical for the development and the progression of cancer and it appears to be involved in numerous steps in tumor progression [27]. Impairment or dysregulation of apoptosis clearly provides a selective advantage to tumoral cells. This resistance could allow the neoplastic cells to evade immunosurveillance as well as environmental changes inherent to tumoral transformation. Indeed, this could explain why, at diagnosis, most tumors have already acquired a certain resistance to apoptosis [28]. This positive selection for apoptosis-resistant tumor cells is accentuated by current therapies (chemo-and radio-therapies), which also use the apoptotic program to kill cancer cells, and thus tumors resistant to these treatments are often highly resistant to apoptosis [29]. Thus, all therapeutic approaches should take into account this innate or acquired resistance in the design of new anti-cancer strategies.

We have addressed the question of the role of apoptosis in tumor progression by using human Bax or Bcl-2 transfected cells and then analyzing tumoral growth in rats. Several clones of the rat glioma A15A5 cells stably transfected with human Bcl-2 or Bax were used (figure 1A). *In vitro *experiments suggest that the expression of the trangenes confer the expected different sensitivities toward apoptosis (figure 1B). We also show that neither the *in vitro *proliferation nor clonogenicity of these cells was affected by the expression of Bcl-2 or Bax (figure 2). Quite remarkably, the expression of Bax suppresses the growth of these tumors in syngenic rats while that of Bcl-2 seems to stimulate the growth (figure 3). The control of tumor growth appeared to be under the control of a specific immune response against tumors. i)Since all tumors proliferated at the same rate in nude mice (figure 4). ii) In the rejected tumors (figure 5), a specific increase in CD8+ cytotoxic lymphocytes, which have the potential to recognize and attack the major histocompatibility complex (MHC) class I-expressing brain cells including tumoral cells [30,31] were detected. iii) Syngenic rats that have received huBax A15A5 cells develop an anti-tumoral reaction against A15A5 cells (figure 6). However, this response was partially occluded by the presence of Bcl-2, a result consistent with the fact that its expression rendered the cells more resistant to apoptosis including that induced by the immune system (figure 1C). However, the accumulation of CD8+ CTL at the site of injection of huBax A15A5 cells could also suggest that induction of cell death in tumors facilitated or triggered a greater specific response. This could explain why an immune response to tumors was specifically observed in animals treated with huBax A15A5 cells (figure 6). On the other hand, the transfection with huBax could modify the phenotype of the rat cells as suggested by previous results [32]. However, MicroArray analysis of huBax A15A5 versus huBcl-2 A15A5 cells did not reveal any changes in the transcriptome of the cells (Cartron and Jézéquel, unpublished observation). Of note, in human glioblastomas expressing Bax ψ, a highly apoptogenic variant of Bax α [17], we observed an accumulation of intra tumoral CD8+ cells when compared to Bax α tumors (Bougras et al., unpublished results).

The apoptosis index has been found to be of significant prognostic significance in patients with high-grade astrocytomas [33]. Our study provides three new findings, which should be considered in immunotherapy: i) the expression of pro-or anti-apoptotic molecules can control the response of the tumor to the immune system during the course of tumor progression (at least in class II). Thus, we suggest that the over-expression of Bcl-2, which often occurs during tumorigenesis could account for the escape from the immune surveillance. ii) Anti-apoptotic mechanisms that often impede the success of treatments could also be an obstacle to immunotherapy. In addition other anti-apoptotic processes such as the existence of soluble decoy receptor that impairs FasL induced-apoptosis in malignant gliomas [34] or the existence of an inactive granzyme [35] could also be involved in the resistance to the immune system. iii) The type of cell death (e.g. apoptosis vs. necrosis) that induces the best stimulation of the immune system is still a matter of controversy but our results suggest that cells sensitized to apoptosis are capable of providing a long lasting and efficient protection against tumoral growth.

Interestingly, a rat model of colon carcinoma has been described in which some clones gave rise to tumors that constantly expanded in the animal to eventually formed metastasis (Pro) while others progress for several days before complete regression (Reg) [36]. The disappearance of the latter clone has been shown to be controlled and to be triggered by the immune system and the transfection of Reg cells by Bcl-2 has been shown to prevent apoptosis and to restore its tumorigenicity [36]. However, the effect of the ectopic expression of Bax in the progressive counterpart cell line Pro was not investigated in this work [36].

Our results show thus for the first time, in the same type of cells, the adverse effects of Bax and Bcl-2 in the antitumoral role of the immune system.

## Conclusion

Taken together, our data provide evidence that BCL-2 family members control tumor growth through their sensitivity to immune-induced cell death and enhancement of the immunogenicity of tumor cells.

## Competing interests

The authors declared that they have no competing interests.

## Abbreviations

Bax: Baxα ; CNS: central nervous system; CTL: cytotoxic T lymphocytes; GBM: Glioblastoma Multiforme; *ic*: intra-cerebral; *sc*: subcutaneous.

## Author's contributions

Gwenola Bougras carried out the phenotyping characterization of the cell lines and in vivo experiments with tumors, Pierre Francois Cartron the molecular genetic studies, Fabien Gautier carried out the experimental studies with apoptotic bodies and Stephane Martin the intracerebral implantation of the rat glioma cell lines. Marité LeCabellec participated in immunohistochemical characterization of the tumors. Marc Grégoire and Khaled Meflah participated in the coordination of the study. Francois M. Vallette conceived of the study, and participated in its design and coordination and drafted the manuscript.

## Pre-publication history

The pre-publication history for this paper can be accessed here:
